# Prognostic significance of nuclear expression of UMP-CMP kinase in triple negative breast cancer patients

**DOI:** 10.1038/srep32027

**Published:** 2016-08-25

**Authors:** Ning Qing Liu, Tommaso De Marchi, Annemieke Timmermans, Anita M. A. C. Trapman-Jansen, Renée Foekens, Maxime P. Look, Marcel Smid, Carolien H. M. van Deurzen, Paul N. Span, Fred C. G. J. Sweep, Julie Benedicte Brask, Vera Timmermans-Wielenga, John A. Foekens, John W. M. Martens, Arzu Umar

**Affiliations:** 1 Department of Medical Oncology, Erasmus MC Cancer Institute, Erasmus University Medical Center, Rotterdam, The Netherlands; 2 Postgraduate School of Molecular Medicine, Erasmus University Medical Center, Rotterdam, The Netherlands; 3 Department of Pathology, Erasmus MC Cancer Institute, Erasmus University Medical Center, Rotterdam, The Netherlands; 4 Department of Radiation Oncology, Radboud University Medical Center, Nijmegen, The Netherlands; 5 Department of Laboratory Medicine, Radboud University Medical Center, Nijmegen, The Netherlands; 6 Department of Pathology, Centre of Diagnostic Investigations, Copenhagen University Hospital, Copenhagen, Denmark

## Abstract

We have previously identified UMP-CMP kinase (CMPK1) as a prognostic marker for triple negative breast cancer (TNBC) by mass spectrometry (MS). In this study we evaluated CMPK1 association to prognosis in an independent set of samples by immunohistochemistry (IHC) and assessed biological pathways associated to its expression through gene set enrichment analysis (GSEA). A total of 461 TNBC paraffin-embedded tissues were collected from different academic hospitals in Europe, incorporated into tissue micro-arrays (TMA), and stained for CMPK1 expression. We also collected gene expression data of 60 samples, which were also present in the TMA, for GSEA correlation analysis. CMPK1 IHC staining showed both cytoplasmic and nuclear components. While cytoplasmic CMPK1 did not show any association to metastasis free survival (MFS), nuclear CMPK1 was associated to poor prognosis independently from other prognostic factors in stratified Cox regression analyses. GSEA correlation analysis of the nuclear CMPK1-stratified gene expression dataset showed a significant enrichment of extracellular matrix (ECM; positive correlation) and cell cycle (negative correlation) associated genes. We have shown here that nuclear CMPK1 is indicative of poor prognosis in TNBCs and that its expression may be related to dysregulation of ECM and cell cycle molecules.

TNBC is a subtype of breast cancer that lacks expression of estrogen receptor (ER), progesterone receptor (PR) and human epidermal growth factor receptor 2 (Her2)^1^. Patients diagnosed with TNBC generally manifest adverse clinical outcome when compared to other breast cancer subtypes and, to date, there is no clinically available targeted therapy for these patients^2^. In this perspective, the majority of TNBC patients receive adjuvant chemotherapy after resection of the primary tumor. Recently, we identified an 11-protein signature that predicts prognosis of TNBC patients by using laser capture microdissection coupled to high resolution MS analysis^3^. While this signature displayed high sensitivity and specificity for predicting disease outcome, introduction of a proteomic workflow as the one used to develop our classifier^4^ into a clinical setting is not yet feasible due to extensive sample preparation and measurement times. In this perspective, an immuno-based (e.g. IHC) assay would constitute a suitable approach to application in clinical diagnostics. CMPK1, as part of the 11-protein signature, was identified as a prognostic marker for lymph-node negative (LNN) and adjuvant systemic chemotherapy naive (ASCN) TNBC patients. In the current study, we used a TMA platform to further investigate the clinical significance of CMPK1 in TNBC patients.

CMPK1 is a 22 kDa enzyme that catalyzes phosphorylation of pyrimidine nucleoside monophosphates, which is essential for *de novo* biosynthesis of pyrimidines^5^,^6^. Also, CMPK1 performs a crucial role in activation of nucleoside analogues used as chemotherapy against human cancers and pathogenic viruses^7^. In a neoplastic setting, decreased expression of CMPK1 mRNA has been associated with 5-fluorouracil resistance of a colorectal cancer cell line HCT-8^8^. Some genetic polymorphisms of CMPK1 have also been reported as a prognostic marker for non-small cell lung cancer^9^ and pancreatic cancer^10^ patients treated with a gemcitabine-based chemotherapy. CMPK1 has been observed in different subcellular locations of HeLa S3 cancer cells, as it predominantly localizes in the cytoplasm but can also enter the nucleus^6^. Subcellular localization of this protein may be important for cancer progression, but no study has yet revealed whether this has prognostic or predictive value in breast cancer. Therefore, further investigation may help to understand the clinical value of this marker, including its subcellular localization.

In this study, we evaluated whether CMPK1 expression in different subcellular compartments is associated to patient prognosis in TNBC patients by IHC analysis of TMA derived from different academic medical centers in Europe. In order to elucidate the molecular pathways associated to subcellular localization of CMPK1, we performed GSEA correlation analysis on a subset of tumors incorporated in the TMA, of which gene expression data was available. A schematic representation of this workflow is shown in Fig. 1.

## Results

### Expression and subcellular localization of CMPK1 in triple negative breast cancer tissues

Analysis of the staining results of an anti-CMPK1 antibody on breast tissues showed that CMPK1 prevalently stained epithelial cells of the mammary gland (normal cells: Fig. S1; carcinoma cells: Fig. S2A–D). When analyzing breast carcinoma tissues, both nuclear and cytoplasmic localization of CMPK1 protein was observed, as previously described in cancer cell lines^6^. Cytoplasmic CMPK1 (cCMPK1) was generally ubiquitous, as the majority of tissues (80.2%) expressed cCMPK1 in more (>) than 70% of carcinoma. Regarding intensity distribution, only a small percentage (9.5%) of tissues was found negative or showed a faint staining (7.0%), while most tissues displayed either weak (33.3%) or moderate (35.5%) cCMPK1 staining. Only 14.7% of cores showed strong cCMPK1 staining intensity (Fig. S2E–F).

On the other hand, nuclear CMPK1 (nCMPK1) staining showed differences in terms of quantity of stained carcinoma cells and staining intensity. Nearly a third (29.7%) of cores did not show any nCMPK1 staining, while positive tissues displayed large heterogeneity in the number of stained tumor cells: 10.6% of tissues showed staining in 1–10% of tumor cells, while 12.8% and 17.2% displayed a number of stained carcinoma cells between 11–25% and 26–50%, respectively. One third (i.e. 29.7%) of tissue cores showed nCMPK1 staining in more than 50% of carcinoma cells (8.8% and 20.9% of the tumors showed expression in 51–70% and >70% of the tumor cells, respectively; Fig. S2E). When assessing nCMPK1 staining intensity distribution, the majority of positive tissues showed weak (16.5%), moderate (23.4%) and strong (24.5%) stainings, with only a small percentage (5.9%) showing faint staining (Fig. S2F). Overall these data show that, while cCMPK1 presents a generally ubiquitous and homogeneous staining, nCMPK1 staining varies widely in terms of stained tumor cells and staining intensity.

### Differences between the TMA datasets

We next compared the three TMA datasets with respect to possible differences in clinical and histopathological parameters (Table 1). The RUMC and CUH cohorts comprised a higher number of postmenopausal women (χ^2^ test *P* < 0.001; Fig. S3A) and a higher median age at diagnosis when compared to the EMC cohort (Kruskal-Wallis test *P* < 0.001; Fig. S3B). There were no differences in pathological tumor size (pT) between the three patient groups (χ^2^ test *P* = 0.085; Fig. S3C). The EMC patient set had relatively more poor grade tumors (Bloom-Richardson grading score^11^; χ^2^ test *P* < 0.001; Fig. S3D), and contained a higher number of lymph node positive patients (χ^2^ test *P* < 0.001; Fig. S3E). Due to the fact that both the RUMC and the CUH cohorts had small numbers of patient samples compared with EMC, we combined the three sets, which skewed the aforementioned observed differences (Table 1).

### Association of CMPK1 subcellular localization to MFS

In order to assess whether nuclear and cytoplasmic CMPK1 stainings measured by IHC were associated to our previous MS findings, a total of 29 samples overlapping with both our TMA and MS sets were analyzed by correlation analysis. No significant correlation was found between cytoplasmic (Spearman r = 0.24; *P* = 0.210; Fig. S4A) or nuclear (Spearman r = −0.04; *P* = 0.824; Fig. S4B) CMPK1 stainings and MS-derived intensity. Taken together, these data support that nCMKP1 nor cCMPK1 analyzed by IHC do not directly relate to total CMPK1 levels measured by high resolution MS.

We next evaluated the prognostic value as a function of both cytoplasmic and nuclear CMPK1 staining. In order to rule out any potential remaining biases due to tissue set of origin, all Cox regression models were stratified according to hospital of origin^12^. Furthermore, for both cytoplasmic and nuclear components of CMPK1 staining, we derived a ‘histo-score’ (see Methods), which resulted from the combination of intensity and quantity categories (histo-score range: 0–20; Table S1). In our stratified Cox regression analyses, patients were stratified based on 4 histo-score categories: 0–5; 6–10; 11–15, and 16–20.

**Figure 2:**
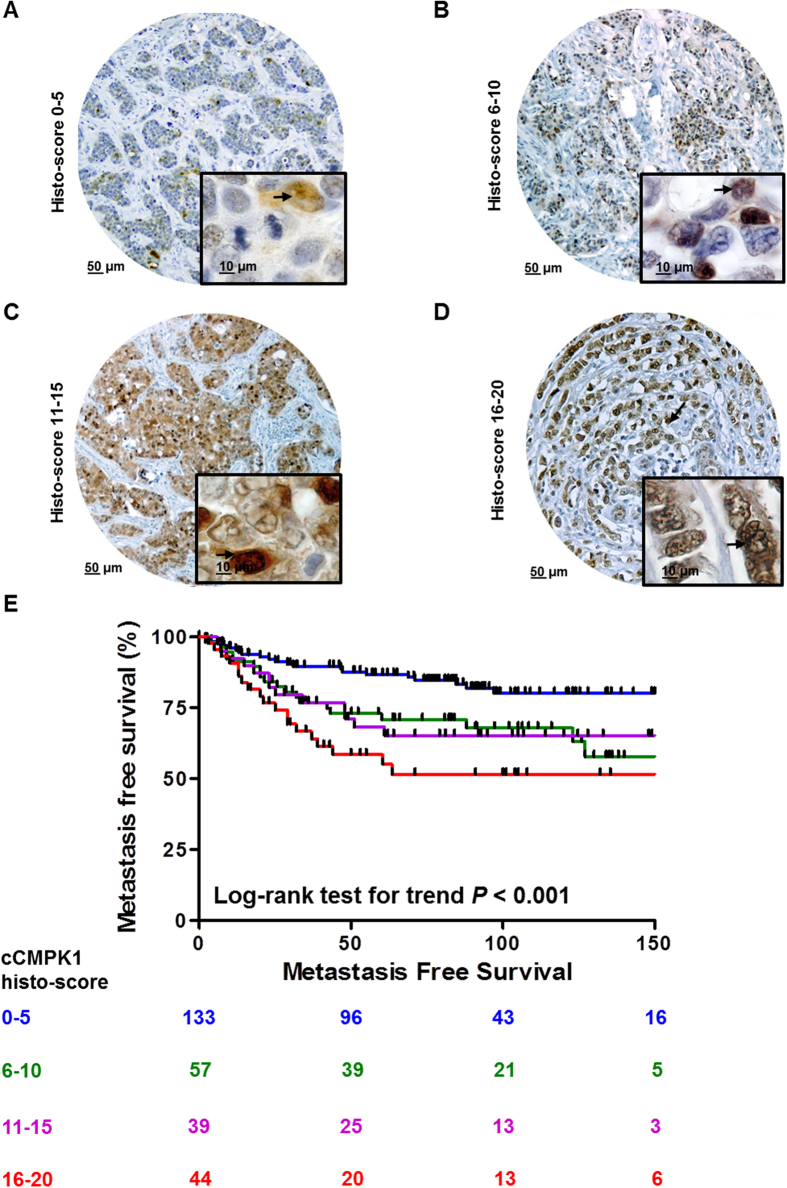
Prognostic significance of nuclear CMPK1 staining. Panels A–D shows breast cancer tissue cores displaying the four nCMPK1 histo-score categories (categories: 0–5, 6–10, 11–15, and 16–20). Kaplan-Meier curves were plotted for each category and Log-rank test for trend was performed (**E**). Acronyms: HR: Hazard Ratio; CI: Confidence Interval.

We first analyzed systemic treatment (chemotherapy) naïve patients with LNN disease only (n = 273; Table 1) to reveal the pure prognostic value of nuclear and cytoplasmic CMPK1. Log-rank test for trend and stratified Cox regression analyses showed that cCMPK1 staining (Fig. S5A–D) was not associated to MFS (Fig. S5E and Table S2). On the other hand, nCMPK1 levels (Fig. 2A–D) were significantly associated with a poor MFS (Fig. 2E) in both univariate and multivariate stratified Cox regression analyses (Table 2). In all Cox models (i.e. univariate and multivariate regression analyses for cCMPK1 and nCMPK1) age, menopausal status, tumor size and differentiation did not show any significant association to MFS.

Having established that nCMPK1 is significantly associated to poor prognosis in treatment naïve patients with LNN disease, we then tested whether chemotherapy treatment influenced the prognostic impact of nCMPK1 as CMPK1 has been connected to chemotherapy response. Importantly, nCMPK1 levels measured by histo-score showed no significant difference between chemotherapy treated and untreated patients (Mann-Whitney *P* = 0.598; Fig. S6). Subsequently, we extended our stratified Cox regression analyses to assess the prognostic value of nCMPK1 in our entire TMA cohort by including chemotherapy treated patients (n = 125; n total = 398). Our analyses showed that, while no significant association between adjuvant chemotherapy treatment and MFS was found, nCMPK1 remained a significant prognostic factor (Fig. S7 and Table S3).

### Analysis of gene expression data associated to nuclear CMPK1 expression

**Figure 3:**
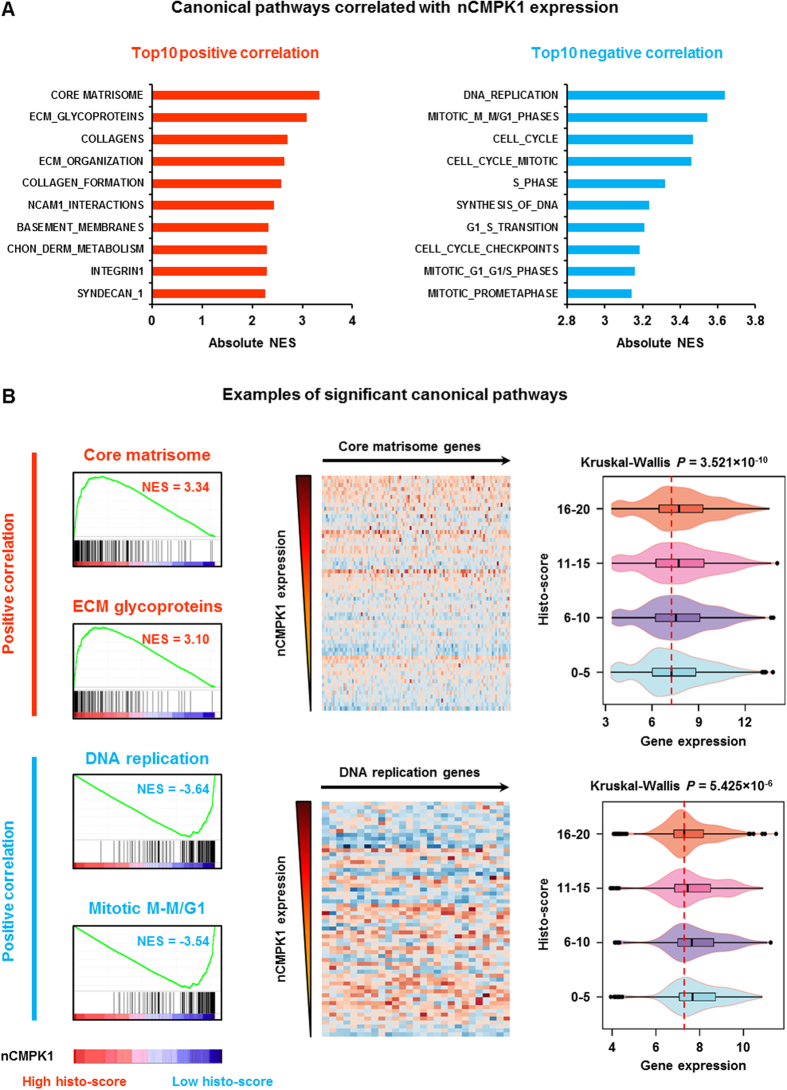
Nuclear CMPK1 (nCMPK1) associated canonical pathways. Panel A displays the summary of top 10 canonical pathways positively (left panel) and negatively (right panel) correlated to nCMPK1 expression (CHON_DERM_METABOLISM: chondroitin sulfate dermatan sulfate metabolism, NES: normalized enrichment score). Panel B displays examples of the pathways associated with nCMPK1 expression. Left panel: enrichment plots of the most significant pathways; Middle panel: heatmaps of the significant genes in ‘core matrisome’ and ‘DNA replication’ pathways; Right panel: expression of the heatmap genes in 4 groups based on nCMPK1 expression.

Having established that only nCMPK1 is related to prognosis in TNBC patients, we sought to elucidate which genes in the entire transcriptome were associated to its expression in order to reveal the putative shared biology with nCMPK1. For this, we collected gene expression data from our previously published dataset^13^ (Gene Expression Omnibus [GEO] ID: GSE2034), and selected samples which overlapped with our TMA cohort (n = 60, n of genes = 10,520). Transcriptome and nCMPK1 histo-scores in the 60 matching samples were correlated by GSEA analysis. We identified that ‘extracellular matrix (ECM) organization’ and ‘DNA replication/cell cycle’ related pathways as the most significant gene sets positively and negatively associated with the nCMPK1 expression, respectively (Fig. 3A). For instance, the ‘core matrisome’ and ‘ECM glycoproteins’ showed the most significant positive correlation with nCMPK1 histo-scores, and we could clearly observe an increased expression of the ‘core matrisome’ genes in the tumors with higher nCMPK1 expression (Fig. 3B, upper panel). Oppositely, the ‘DNA replication’ and ‘mitotic M-M/G1’ genes were the most significant gene sets negatively correlated with nCMPK1 expression, and higher expression of the ‘DNA replication’ genes were observed in the tumors with less nCMPK1 expression (Fig. 3B, lower panel). Taken together, these data indicate that nCMPK1 levels are associated to ECM and cell cycle related molecules.

## Discussion

The predictive and prognostic value of CMPK1 mRNA expression and genetic polymorphisms have been described in colorectal tumors^8^, non-small cell lung carcinoma^9^, and pancreatic cancer patients who received pyrimidine nucleoside analogue-based chemotherapies as first line treatment^10^. Also, CMPK1 has been shown to localize both in the nucleus and cytoplasm of cancer cells^6^, though the clinical relationship of this finding in TNBC has not been investigated. In the current study, we have investigated the association of CMPK1 subcellular localization with prognosis in TNBC patients.

IHC staining of TNBC tissues included in a TMA showed that CMPK1 was expressed both in the nucleus as well as in the cytoplasm of breast carcinoma cells, as it was also discovered for another marker (i.e. FTH1) belonging to the same signature^3^,^14^. While the cytoplasmic component appeared to be ubiquitous and homogeneous, as staining intensity was generally weak or moderate and present in the majority (>70%) of tumor cells, nCMPK1 displayed a higher degree of heterogeneity in terms of expression and number of stained tumor cells. The assessment of the clinical relevance of both cCMPK1 and nCMPK1 expression (derived from histo-score calculations) using stratified Cox regression models showed that cCMPK1 levels were not associated with prognosis of TNBCs, probably due to its ubiquitous expression in breast carcinomas included in the TMA. On the other hand, nCMPK1 expression was found significantly associated with poor prognosis. In fact, we not only established that nCMPK1 is indicative of poor prognosis in a chemotherapy untreated group, thus reflecting the natural course of the disease (i.e. LNN and chemotherapy naive), but we also showed that the prognostic power is independent of clinical and histopathological factors such as age, menopausal status, tumor size, grade, presence of lymph node metastases and adjuvant chemotherapy treatment. Furthermore, the fact that nCMPK1 is associated to poor prognosis, opposite of what has been shown for total CMPK1 expression as measured by MS, may indicate a differential role for this kinase depending on its subcellular localization. This apparent discrepancy with our previous MS-based findings may be due to the fact that cCMPK1 staining, as represented in the majority of IHC stained tumor cells, likely better represented the total CMPK1 expression as measured by MS in our previous study, though no correlation between any IHC CMPK1 stainings and MS data was found. This may be ascribed to the fact that, on one hand, MS provided an accurate quantitative measure of protein levels but cannot differentiate between nuclear and cytoplasmic CMPK1. On the other hand, IHC is a semi-quantitative technique that cannot very accurately assess target protein levels due to limitations in chromogenic signal quantification, as it has also been reported for other (breast cancer) markers^15^.

After having established clinical significance for nCMPK1 in TNBC, we explored the biology correlated to nCMPK1 expression through pathway analysis on a subset of samples for which both gene expression and IHC data were available. CMPK1 is involved in cytidine metabolism and incorporation during DNA replication. Two recent studies have shown that CMPK1 is crucial for the incorporation of deoxycytidine (dC), and prevents the introduction of modified cytosines (5-methyl-2′deoxycytidine/5mdC, 5-hydroxymethyl-2′deoxycytidine/5hmdC and 5-formy-2′deoxycytidine/5fdC) during the DNA replication process^16^. Correct dC integration prevents oxidative damage on the DNA of tumor cells introduced by incorporation of 5hmdC and is therefore essential to maintain genomic integrity during the normal cell cycle. In line with this mechanism, in the current study we identified nCMPK1 as a negative cell cycle regulator. This observation is contradictive in respect to the knowledge that fast cell cycle allows high proliferation and tumor malignancy. However, nCMPK1 may contribute to cancer cell survival and malignancy in a unique manner: Oxidative stress, as a hallmark for cancer^17^, causes accumulation of modified cytotoxic cytosines in tumor cells, which can be integrated into DNA replication by cytidine deaminase (CDA). The nCMPK1 components block the incorporation path of the modified cytosines and only allows dC incorporation during DNA replication^16^. Therefore, increased nCMPK1 may force the cell into a dC incorporation flux while, at the same time, slows down the cell cycle and consequently reduces the incorporation rate of modified cytosines caused by rapid cell cycling. On the other hand, oxidative stress results in alteration of tumor microenvironment, which contributes to the reorganization of the extracellular matrix (ECM) and provides a favorable metastatic environment for the tumor cells^18^. Hence, it is not unexpected that the gene sets related to ECM organization were positively associated with nCMPK1 expression.

In conclusion, we report that nuclear expression of CMPK1, as measured by IHC, is associated with poor prognosis in TNBC independently of clinical and histopathological factors, and it is associated to ECM and cell cycle molecules dysregulation. Further studies are needed though to establish the causality between these mechanisms and to possibly derive new therapeutic targets.

## Methods

### Primary breast cancer tissues

A total of 627 primary breast cancer tissues were collected from Erasmus Medical Center (EMC; n = 412), Radboud University Medical Center (RUMC; n = 129), and Copenhagen University Hospital (CUH; n = 86), and were used to construct three separate TMAs. Expression of ER, PR and Her2 proteins was determined by IHC staining, and fluorescence in situ hybridization was performed when staining of Her2 was scored as 2+ (i.e. uncertain amplification), to assess possible amplification of this gene. Samples were excluded from further analysis when tissues had unclear expression of ER, PR or Her2, no information on lymph-node status and/or adjuvant systemic chemotherapy, and/or poor quality of CMPK1 staining (defined as: heterogeneous staining between cores belonging to the same specimen and/or lack of triplicate staining due to lack of at least one core), resulting in a final number of 267 (EMC), 48 (RUMC), and 83 (CUH) tissues for data analysis, respectively. Out of a total of 398 samples included in the TMA, Clinical and histopathological features of these tissues are summarized in Table 1.

This study was approved by the Medical Ethics Committee of the EMC, The Netherlands (MEC 02.953) and was performed in accordance to the Code of Conduct of the Federation of Medical Scientific Societies in The Netherlands, and wherever possible we adhered to the Reporting Recommendations for Tumor Marker Prognostic Studies (REMARK)^19^. Informed consent was obtained from all patients.

### Tissue micro-array

The TMAs were prepared using an ATA 27 (Beecher Instruments, Sun Prairie, WI, USA). For every tissue, a specialized breast pathologist marked the tumor area, from which three different cores (diameter: 0.6 mm) were taken as biological replicates and transferred in a TMA recipient block. Tissue cores of 0.6 mm were taken from each tissue paraffin block and transferred in triplicate into a TMA recipient block. Stained TMA slides were digitalized and analyzed using Slidepath software (Leica Microsystems, Solms, Germany).

### IHC staining

Sections of 4 μm of the three above mentioned TMAs were incubated for 1 hour at room temperature with CMPK1 antibody at a dilution of 1:300 against the protein (mouse monoclonal, clone 1D7; Lifespan Bioscience, Seattle, WA, USA). Antigen retrieval was performed prior to antibody incubation by heating the slides for 40 min at 95 °C and washing with Dako antigen retrieval solution (pH = 6) (DakoCytomation, Carpinteria, CA, USA) when the slides were cooled down to room temperature. Staining was visualized by anti-mouse EnVision+^®^ System-HRP (DAB) (DakoCytomation, Carpinteria, CA, USA). CMPK1 staining was separately scored in both percentage of positive invasive breast carcinoma cells and staining intensity by two independent observers. Six categories were scored for the percentage of positive invasive tumor cells: 0%, 1–10%, 11–25%, 26–50%, 51–70%, and >70%. All cores present on the three TMAs were scored by an experienced researcher in a blind manner. Staining score of triplicate cores were validated by a second experienced researcher, who was extensively trained by a specialized breast pathologist.

### Data analysis

Differences between centers of origin were evaluated prior CMPK1 IHC staining analysis. Differences in patient menopausal status, tumor size, tumor differentiation and number of number of positive lymph nodes were assessed by χ^2^ test, while Kruskal-Wallis test was performed to assess differences in patient age.

In order to derive a more accurate measurement of CMPK1 expression as measured by IHC, intensity and quantity categories were transformed into numerical values (Table S1) and a ‘histo-score’ (as reported in^15^) was calculated for both the cytoplasmic and nuclear staining components with the following formula:


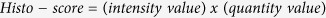


Nuclear and cytoplasmic CMPK1 histo-score distributions were divided into four categories: 0–5, 6–10, 11–15, and 16–20 (Fig. 2A–D and Fig. S4A–D). Center of origin stratified univariate and multivariate Cox regression analyses were performed using Stata software (version 12.0). Clinical and histo-pathological characteristics included in Cox regression multivariate models were: age at diagnosis (categories: <40, 41–55, 56–70, >70), menopausal status (categories: premenopausal, postmenopausal), tumor size (categories: pT1, pT2 + pTx, pT3 + pT4), and tumor grade (Bloom-Richardson; categories: good, moderate, poor). Survival curves were plotted and Log-rank test for trend was performed. In order to test whether nCMPK1 histo-score showed an association to chemotherapy treatment, we assessed whether a difference in nCMPK1 levels between chemotherapy treated and untreated patients existed by Mann-Whitney test. Previously reported MS data (ProteomeXchange identifier: PXD000260) of samples also included in the TMA were collected and Log10 CMPK1 intensity levels were compared with matching nuclear and cytoplasmic histo-scores. Spearman correlation analysis was performed to test the association between CMPK1 IHC and MS levels. Kruskal-Wallis, χ^2^ test, Log-rank test for trend, and Spearman correlation analyses were performed in GraphPad (version 5.1).

Affymetrix gene expression data of 60 samples that matched with the TNBC tissues on the TMA (deposited in GEO with dataset identifier: GSE2034) were collected and stratified according to nCMPK1 IHC stainings (histo-score categories: 0–5, 6–10, 11–15, 16–20). GSEA was performed on this set of samples to identify gene sets correlated with nCMPK1 IHC expression using a canonical pathway Molecular Signatures Database (MSigDB) (version 5.1)^20^. False discovery rates (FDRs) of enriched pathways were estimated based on 1,000 time permutation on defined phenotypes with fixed 149 seeds. Multiple probes assigned to the same gene were collapsed into single gene expression using the probes with the highest expression value for every tested sample. Heat-maps and violin plots were generated using Bioconductor (version 3.0) package ‘made4’ and ‘ggplot2’, respectively.

## Additional Information

**How to cite this article**: Liu, N. Q. *et al*. Prognostic significance of nuclear expression of UMP-CMP kinase in triple negative breast cancer patients. *Sci. Rep.* **6**, 32027; doi: 10.1038/srep32027 (2016).

## Supplementary Material

Supplementary Information

## Figures and Tables

**Figure 1:**
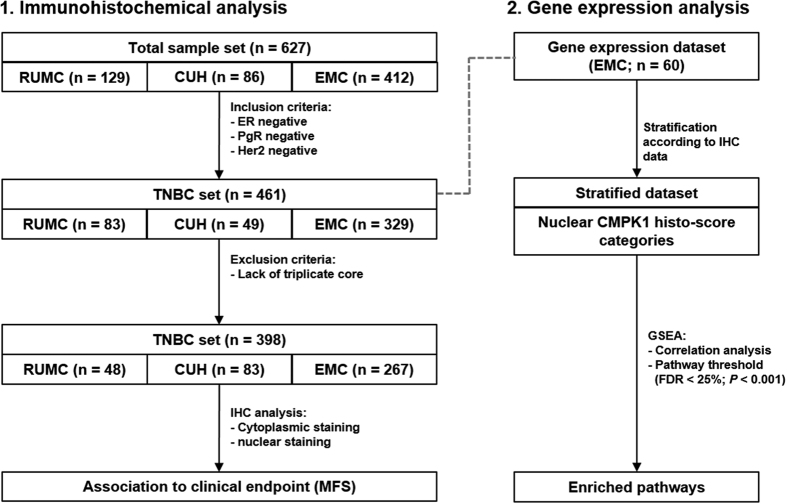
Schematic representation of experimental workflow.

**Table 1:** Clinical and histopathological characteristics of patients included in the TMAs.

	RUMC*	CUH*	EMC*	Total*
Total n of patients	48 (100.0)	83 (100.0)	267 (100.0)	398 (100.0)
Age (years)				
≤40	7 (14.6)	12 (14.5)	65 (24.3)	84 (21.1)
41–55	18 (37.5)	22 (26.5)	113 (42.3)	153 (38.4)
56–70	19 (39.6)	29 (34.9)	67 (25.1)	115 (28.9)
>70	4 (8.3)	20 (24.1)	22 (8.3)	46 (11.6)
Menopausal status				
Premenopausal	18 (37.5)	24 (28.9)	155 (58.0)	197 (49.5)
Postmenopausal	30 (62.5)	59 (71.1)	112 (42.0)	201 (50.5)
Tumor size				
pT1	22 (45.8)	49 (59.1)	107 (40.1)	178 (44.7)
pT2 + pTx	24 (50.0)	30 (36.1)	147 (55.0)	201 (50.5)
pT3 + pT4	2 (4.2)	4 (4.8)	13 (4.9)	19 (4.8)
Tumor Grade				
Good	0 (0.0)	2 (2.4)	4 (1.5)	6 (1.5)
Moderate	5 (10.4)	8 (9.6)	44 (16.5)	57 (14.3)
Poor	32 (66.7)	54 (65.1)	213 (79.8)	299 (75.1)
Unknown	11 (22.9)	19 (22.9)	6 (2.2)	36 (9.1)
Lymph node positivity				
0	46 (95.8)	83 (100.0)	202 (75.6)	331 (83.2)
1 to 3	2 (4.2)	0 (0.0)	44 (16.5)	46 (11.6)
>3	0 (0.0)	0 (0.0)	21 (7.9)	21 (5.2)
Adjuvant chemotherapy				
Yes	2 (4.2)	40 (48.2)	83 (31.1)	125 (31.4)
No	46 (95.8)	43 (51.8)	184 (68.9)	273 (68.6)

^*^Data are displayed as number (percentage).

Acronyms: CUH: Copenhagen University Hospital; EMC: Erasmus University Medical Center; RUMC: Radboud University Medical Center.

**Table 2:** Stratified Cox regression analyses for the association of nCMPK1 to MFS in lymph-node negative and adjuvant therapy naïve patients.

	n of patients	Univariate			Multivariate		
		HR	95% CI	*P*	HR	95% CI	*P*
nCMPK1 histo-score							
0–5	133	1.00			1.00		
6–10	57	2.26	1.22 to 4.20	0.009	2.46	1.30 to 4.66	0.006
11–15	39	1.98	0.99 to 3.96	0.054	1.91	0.90 to 4.06	0.090
16–20	44	2.74	1.48 to 5.07	0.001	2.66	1.39 to 5.08	0.003
Age (years)							
<40	51	1.00			1.00		
41–55	86	0.86	0.48 to 1.56	0.634	0.89	0.46 to 1.70	0.724
56–70	90	0.82	0.45 to 1.48	0.511	1.05	0.36 to 3.10	0.921
>70	46	0.46	0.17 to 1.25	0.130	0.51	0.13 to 1.98	0.330
Menopausal status							
Premenopausal	115	1.00			1.00		
Postmenopausal	158	0.74	0.47 to 1.18	0.213	0.86	0.35 to 2.14	0.747
Tumor size							
pT1	119	1.00			1.00		
pT2 + pTx	143	1.11	0.70 to 1.76	0.665	1.35	0.82 to 2.21	0.238
pT3 + pT4	11	0.51	0.07 to 3.78	0.515	0.83	0.11 to 6.34	0.861
Tumor Grade							
Good	3	1.00			1.00		
Moderate	38	0.48	0.11 to 2.11	0.329	0.42	0.08 to 2.16	0.299
Poor	206	0.29	0.07 to 1.19	0.087	0.31	0.06 to 1.53	0.152
Unknown	26	0.15	0.02 to 1.13	0.066	0.12	0.01 to 0.99	0.049

Acronyms: CI: confidence interval; HR: hazard ratio.
