# Supplementary material for: Prognostic significance of nuclear expression of UMP-CMP kinase in triple negative breast cancer patients
Source: Sci Rep. 2016 Aug 25;6:32027. doi: 10.1038/srep32027 (PMC4997324; doi:10.1038/srep32027)

Title: Prognostic significance of nuclear expression of UMP-CMP kinase in triple negative breast cancer patients

Ning Qing Liu[1,7,8,*,†], Tommaso De Marchi[1,7,*,†], Annemieke Timmermans[1], Anita M.A.C. Trapman-Jansen[1], Renée Foekens[1], Maxime P. Look[1], Marcel Smid[1], Carolien H.M. van Deurzen[2], Paul N. Span[3], Fred C.G.J. Sweep[4], Julie Benedicte Brask[5], Vera Timmermans-Wielenga[5], John A. Foekens[1], John W.M. Martens[1], and Arzu Umar[1,†].

1. Department of Medical Oncology, Erasmus MC Cancer Institute, Erasmus University Medical Center, Rotterdam, The Netherlands.

2. Department of Pathology, Erasmus MC Cancer Institute, Erasmus University Medical Center, Rotterdam, The Netherlands.

3. Department of Radiation Oncology, Radboud University Medical Center, Nijmegen, The Netherlands.

4. Department of Laboratory Medicine, Radboud University Medical Center, Nijmegen, The Netherlands.

5. Department of Pathology, Centre of Diagnostic Investigations, Copenhagen University Hospital, Copenhagen, Denmark.

7. Postgraduate School of Molecular Medicine, Erasmus University Medical Center, Rotterdam, The Netherlands.

8. Current address: Department of Molecular Biology, Faculty of Science, Nijmegen Centre for Molecular Life Sciences, Radboud University, Nijmegen, The Netherlands.

* These authors equally contributed to this study.

† Corresponding authors

Supplementary Figures Legends:

Figure S1. CMPK1 expression in the normal mammary gland.

Panels display CMPK1 staining in normal mammary acini (A) and ducts (B). CMPK1 was expressed both in the epithelial as well as in the basal cell layers of the mammary gland.

Figure S2. CMPK1 immunohistochemical stainings and their frequencies in the TMA sets.

IHC staining showed that CMPK1 protein is localized in both the nucleus and the cytoplasm. Panels A-D display examples of such stainings. Cytoplasmic and nuclear stainings differed greatly in terms of both quantity and intensity of staining, with the cytoplasmic one being generally ubiquitous and homogeneous.

Acronyms: IHC: immunohistochemistry.

Figure S3. Assessment of differences in clinical and histopathological parameters between the TMA datasets.

Differences between EMC, RUMC and CUH sets were evaluated. Significant differences were observed in the number of pre- and post-menopausal women between sets, with the RUMC and CUH cohort being enriched of the latter (χ^2 test $P < 0.001$; A). Furthermore, CUH and RUMC patients were generally older than EMC patients (Kruskal-Wallis $P < 0.001$; B). No difference in tumor size was observed between the sets (χ^2 test $P = 0.085$; C), while a significant difference was observed for tumor grade (χ^2 test $P < 0.001$; D) and number of positive lymph nodes (χ^2 test $P < 0.001$; E).

Acronyms: CUH: Copenhagen University Hospital; EMC: Erasmus University Medical Center; RUMC: Radboud University Medical Center.

Figure S4. Association between IHC and MS data.

A total of 29 tumors were comprised in both our TMA and MS cohorts. Spearman correlation analysis was performed in order to evaluate the association between CMPK1 IHC stainings (panel A: cytoplasmic; panel B: nuclear) and high resolution MS measured intensity.

Acronyms: IHC: immunohistochemistry; MS: mass spectrometry; TMA: tissue micro-array.

Figure S5. Cytoplasmic CMPK1 stainings stratified by histo-score and their association to MFS.

Panels A-D represent the four histo-score categories (i.e. 0-5, 6-10, 11-15, and 16-20) in which the cytoplasmic CMPK1 histo-score was divided. Survival curves of patients whose tumors were groups in each of the aforementioned histo-score categories are shown in panel E.

Figure S6. Difference in nCMPK1 levels between chemotherapy treated and untreated patients. Differences in nCMPK1 histo-score levels between adjuvant systemic chemotherapy treated and naïve patients were assessed by Mann-Whitney test.

Figure S7. Nuclear CMPK1 stainings stratified by histo-score and their association to MFS in all patients. All patients (n = 398) included in our study were stratified according to nCMPK1 histo-score categories. Survival curves were plotted and differences were analyzed by Log-rank test for trend.

Supplementary tables:

Table S1. Histo-score calculation for nuclear and cytoplasmic CMPK1.

	Categories					
**Staining intensity**	Negative	Faint	Weak	Moderate	Strong	
**Histo score intensity**	0	1	2	3	4	
**Staining quantity**	0%	1-10%	11-25%	26-50%	51-75%	>75%
**Histo score quantity**	0	1	2	3	4	5

Table S2. Stratified Cox regression analyses for the association of cCMPK1 to MFS.

	n of patients	Univariate			Multivariate		
		**HR**	**95% CI**	***P***	**HR**	**95% CI**	***P***
**cCMPK1 histo-score**							
**0-5**	48	1.00			1.00		
**6-10**	90	0.66	0.28 to 1.55	0.342	0.74	0.30 to 1.80	0.505
**11-15**	96	0.68	0.29 to 1.58	0.367	0.75	0.31 to 1.80	0.518
**16-20**	39	0.57	0.22 to 1.51	0.261	0.71	0.26 to 1.96	0.511
**Age (years)**							
**< 40**	51	1.00			1.00		
**41-55**	86	0.86	0.48 to 1.56	0.634	0.95	0.50 to 1.81	0.875
**56-70**	90	0.82	0.45 to 1.48	0.511	1.03	0.37 to 2.88	0.958
**> 70**	46	0.46	0.17 to 1.25	0.130	0.53	0.26 to 2.05	0.359
**Menopausal status**							
**Premenopausal**	115	1.00			1.00		
**Postmenopausal**	158	0.74	0.47 to 1.18	0.213	0.81	0.34 to 1.92	0.640
**Tumor size**							
**pT1**	119	1.00			1.00		
**pT2 + pTx**	143	1.11	0.70 to 1.76	0.665	1.24	0.76 to 2.02	0.391
**pT3 + pT4**	11	0.51	0.07 to 3.78	0.515	0.78	0.10 to 5.87	0.808
**Tumor Grade**							
**Good**	3	1.00			1.00		
**Moderate**	38	0.48	0.11 to 2.11	0.329	0.48	0.10 to 2.35	0.370
**Poor**	206	0.29	0.07 to 1.19	0.087	0.29	0.06 to 1.33	0.112
**Unknown**	26	0.15	0.02 to 1.13	0.066	0.15	0.02 to 1.17	0.071

Acronyms: CI: confidence interval; HR: hazard ratio.

Table S3. Stratified Cox regression analyses for the association of nCMPK1 to MFS in all patients.

	n of patients	Univariate			Multivariate		
		HR	95% CI	*P*	HR	95% CI	*P*
**nCMPK1 histo-score**							
**0-5**	195	1.00			1.00		
**6-10**	88	1.90	0.15 to 3.12	0.012	2.08	1.25 to 3.46	0.005
**11-15**	51	1.75	0.98 to 3.10	0.057	1.87	0.99 to 3.51	0.050
**16-20**	64	2.36	1.42 to 3.90	0.001	2.37	1.40 to 4.02	0.001
**Age (years)**							
**< 40**	84	1.00			1.00		
**41-55**	153	0.82	0.52 to 1.30	0.402	0.93	0.57 to 1.51	0.770
**56-70**	115	0.71	0.43 to 1.20	0.202	1.29	0.51 to 3.25	0.583
**> 70**	46	0.43	0.17 to 1.11	0.082	0.64	0.18 to 2.21	0.479
**Menopausal status**							
**Premenopausal**	197	1.00			1.00		
**Postmenopausal**	201	0.64	0.43 to 0.95	0.027	0.644	0.30 to 1.40	0.268
**Tumor size**							
**pT1**	178	1.00			1.00		
**pT2 + pTx**	201	1.46	0.98 to 2.19	0.063	1.57	1.03 to 2.39	0.037
**pT3 + pT4**	19	1.46	0.57 to 3.72	0.423	1.20	0.44 to 3.30	0.717
**Tumor Grade**							
**Good**	6	1.00			1.00		
**Moderate**	57	0.29	0.10 to 0.85	0.025	0.27	0.08 to 0.92	0.036
**Poor**	299	0.23	0.85 to 0.65	0.005	0.27	0.09 to 0.87	0.029
**Unknown**	36	0.08	0.01 to 0.48	0.005	0.08	0.01 to 0.49	0.006
**Lymph node positivity**							
**0**	331	1.00			1.00		
**1 to 3**	46	1.47	0.90 to 2.40	0.118	2.20	0.68 to 7.10	0.186
**> 3**	21	1.58	0.79 to 3.17	0.197	2.58	0.72 to 9.24	0.144
**Adjuvant chemotherapy**							
**Yes**	125	1.00			1.00		
**No**	273	1.18	0.78 to 1.78	0.441	0.52	0.18 to 1.56	0.245

Acronyms: CI: confidence interval; HR: hazard ratio.

Supplementary Figures:

Figure S1. CMPK1 expression in the normal mammary gland.

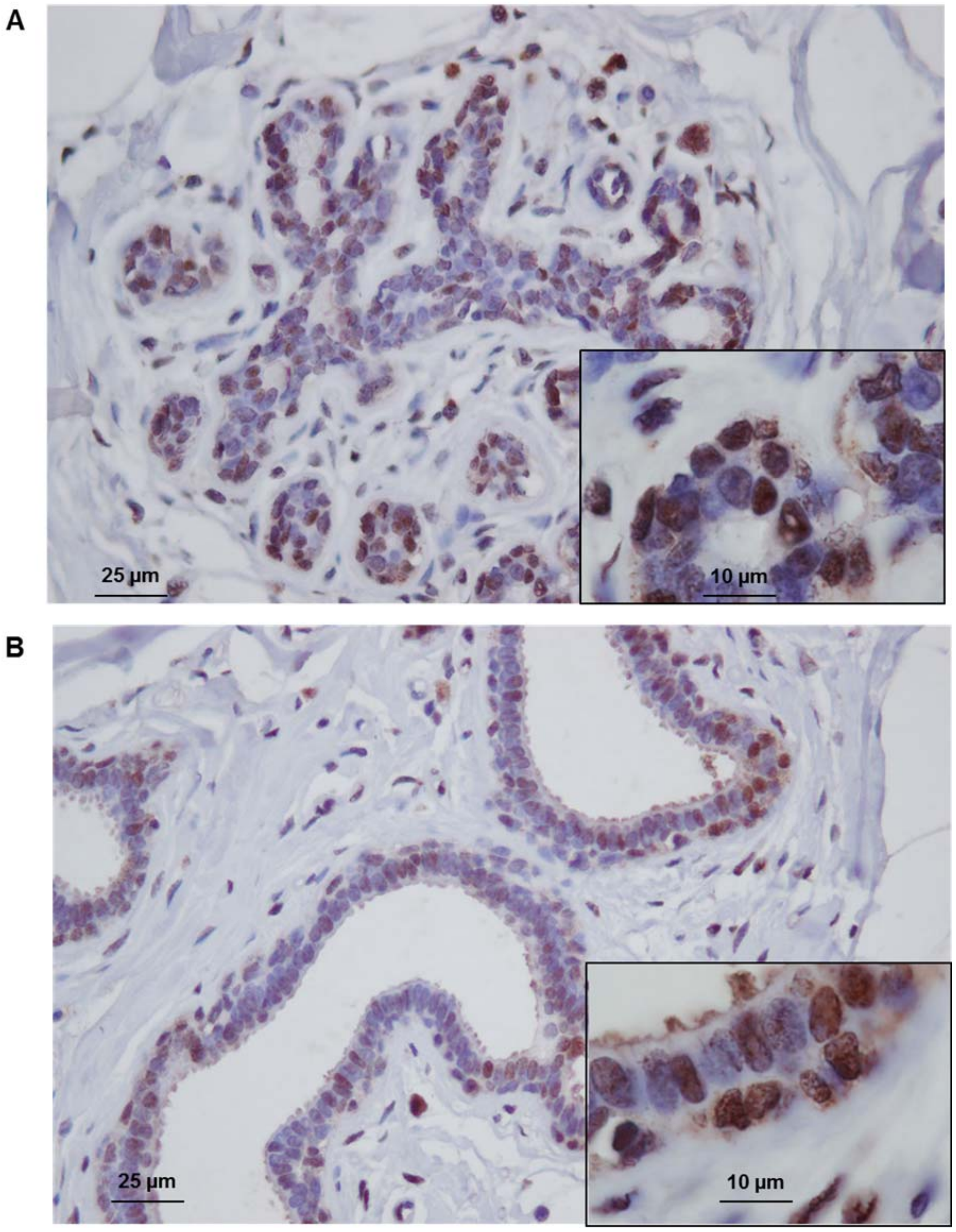

Figure S2. CMPK1 immunohistochemical stainings and their frequencies in the TMA sets.

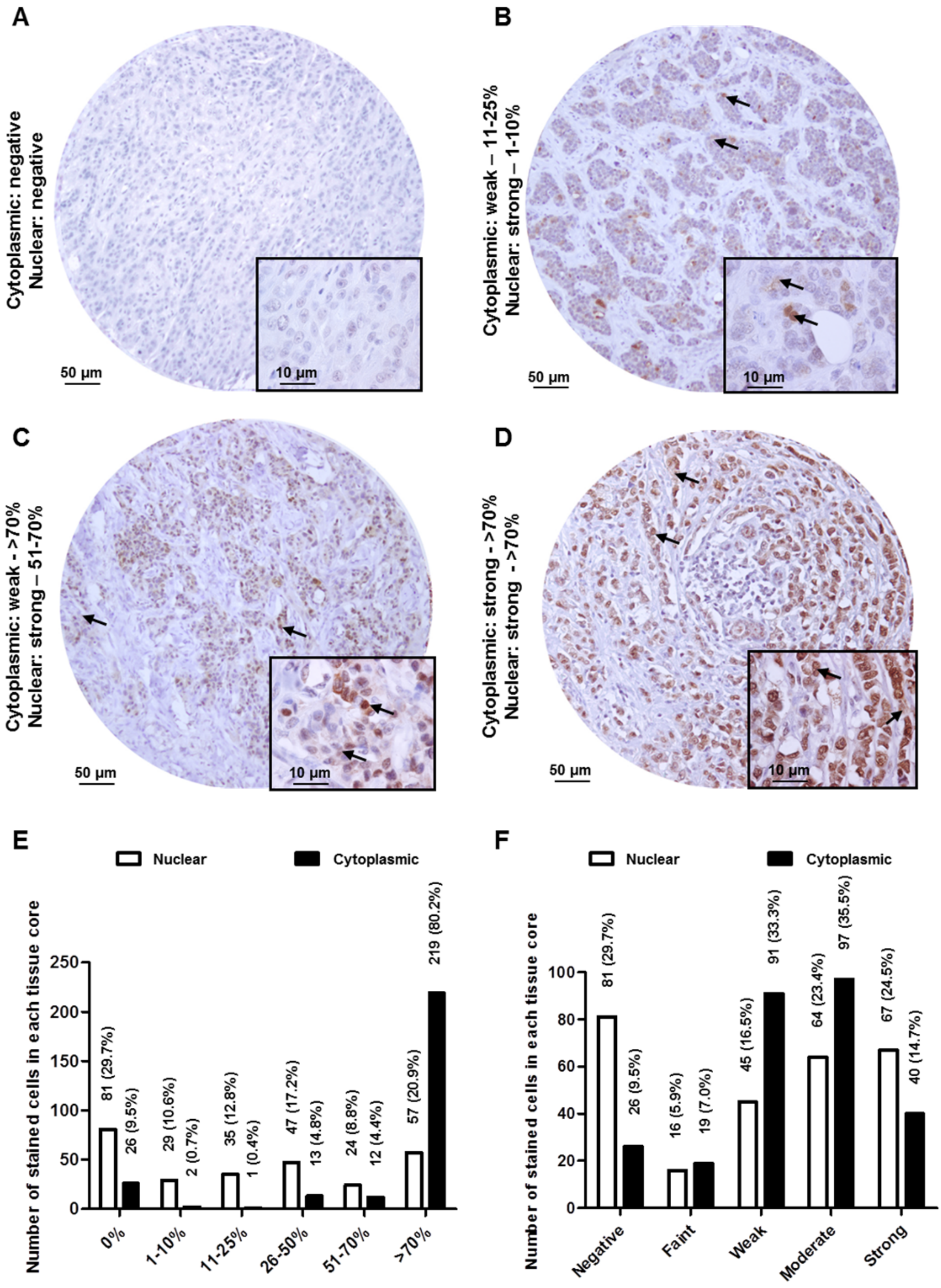

Figure S3. Assessment of differences in clinical and histopathological parameters between the TMA datasets.

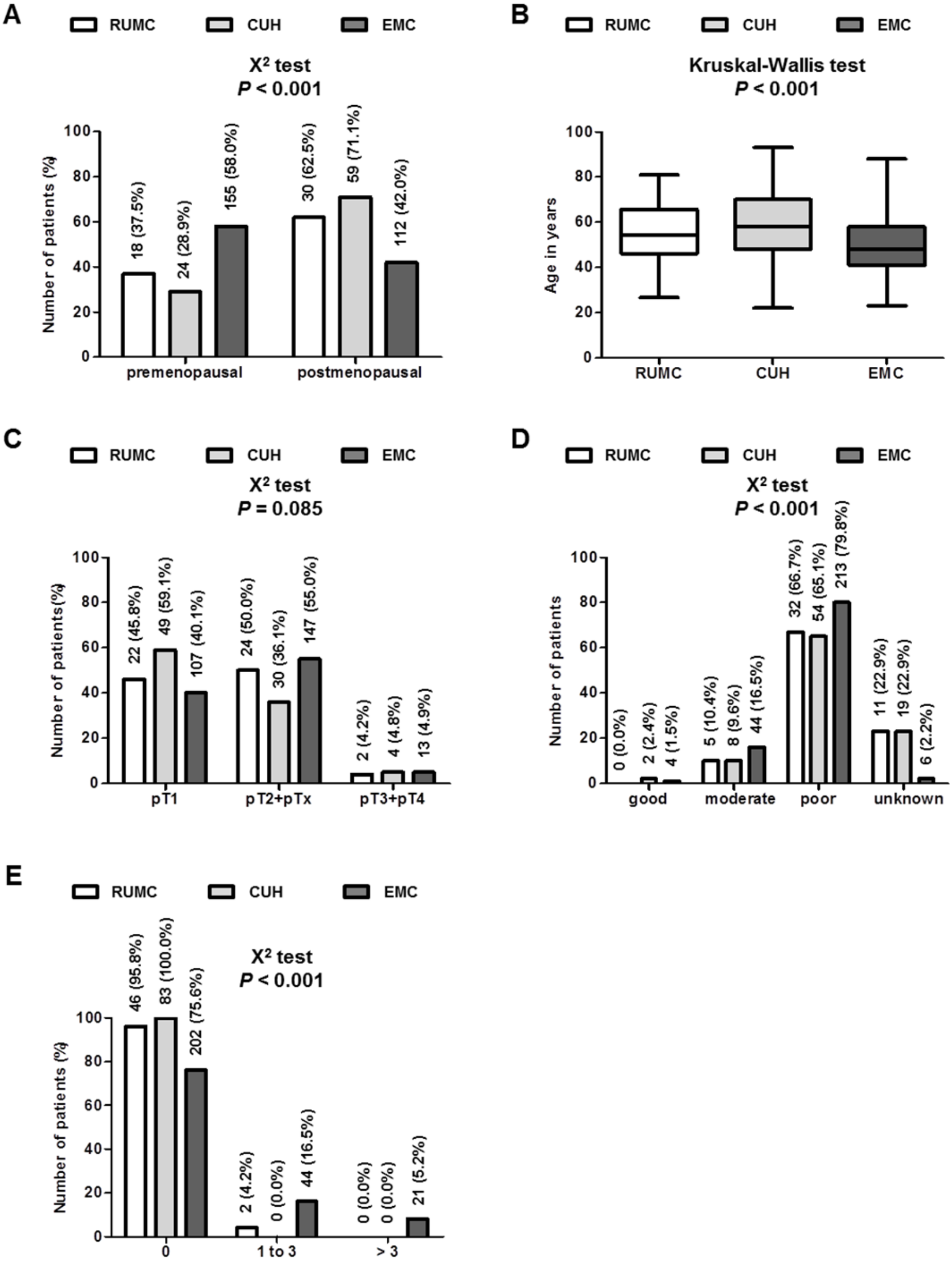

Figure S4. Association between IHC and MS data.

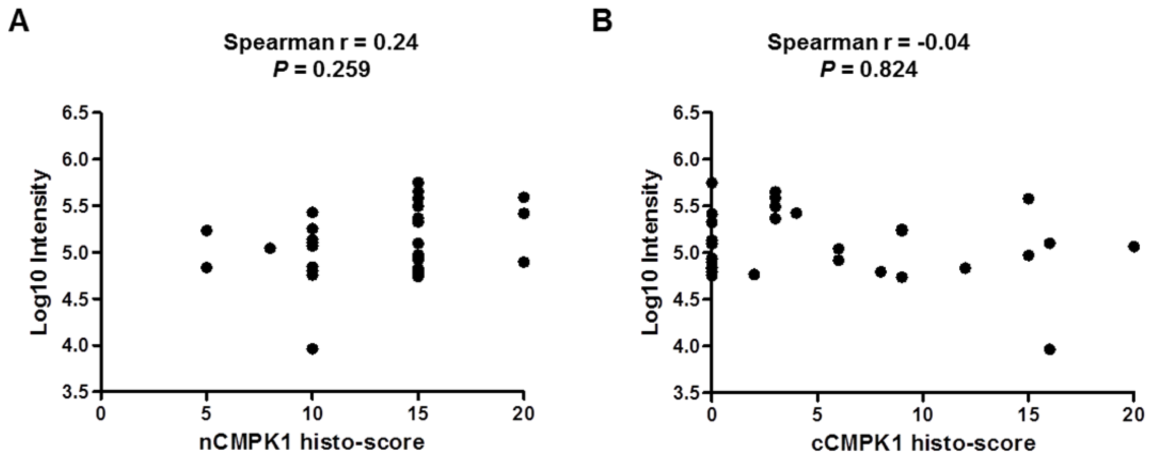

Figure S5. Cytoplasmic CMPK1 stainings stratified by histo-score and their association to MFS.

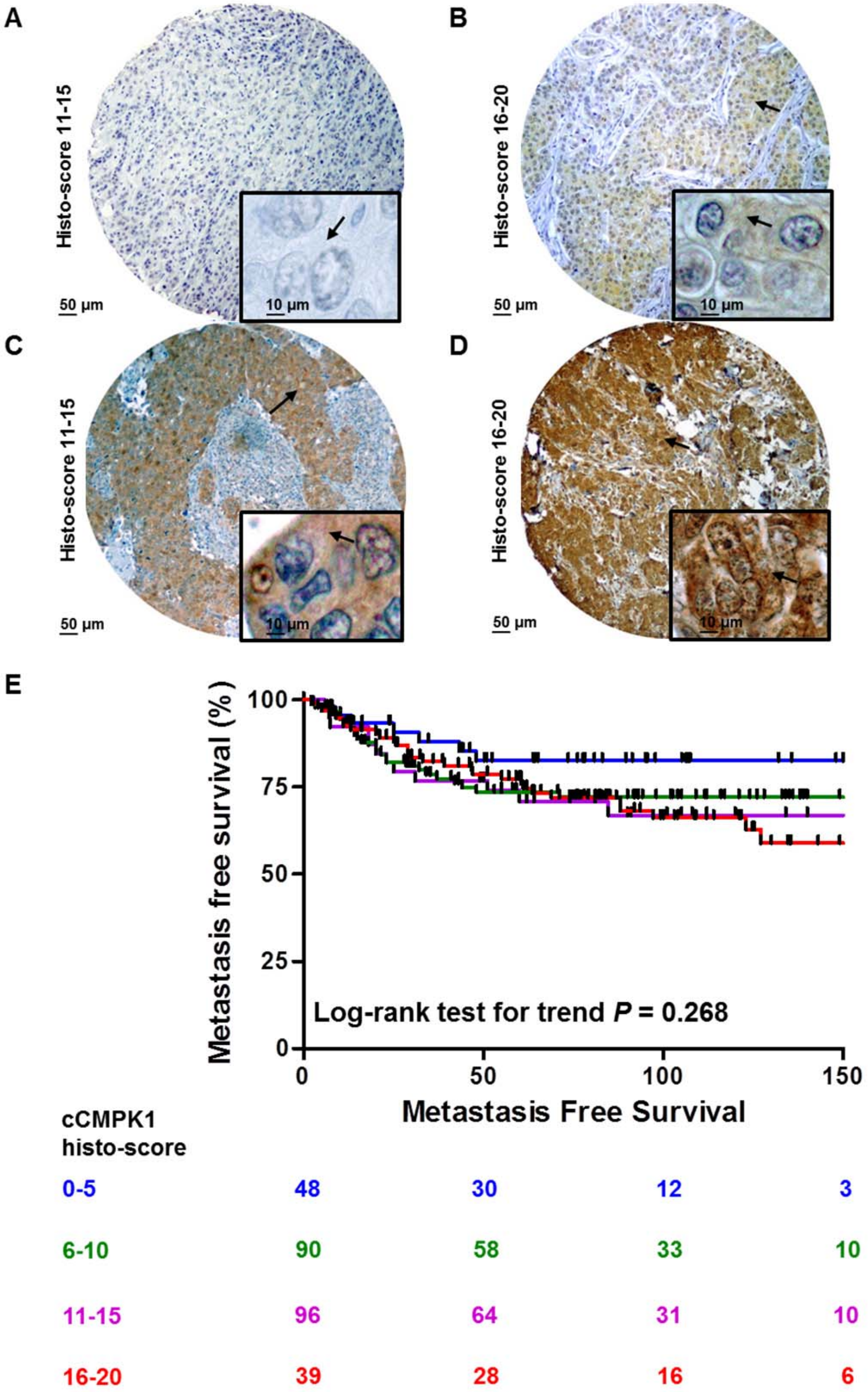

Figure S6. Difference in nCMPK1 levels between chemotherapy treated and untreated patients.

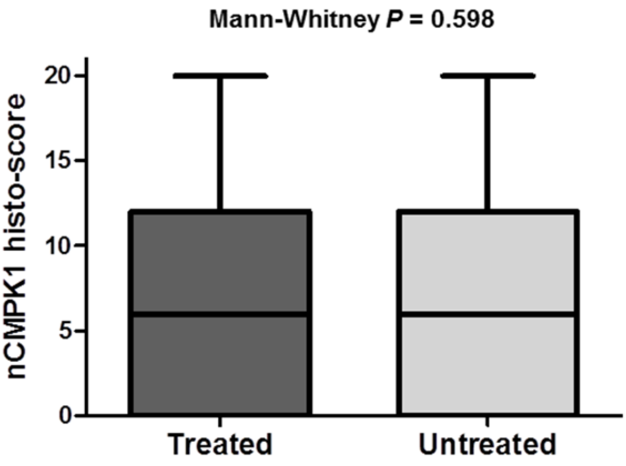

Figure S7. Nuclear CMPK1 stainings stratified by histo-score and their association to MFS in all patients.

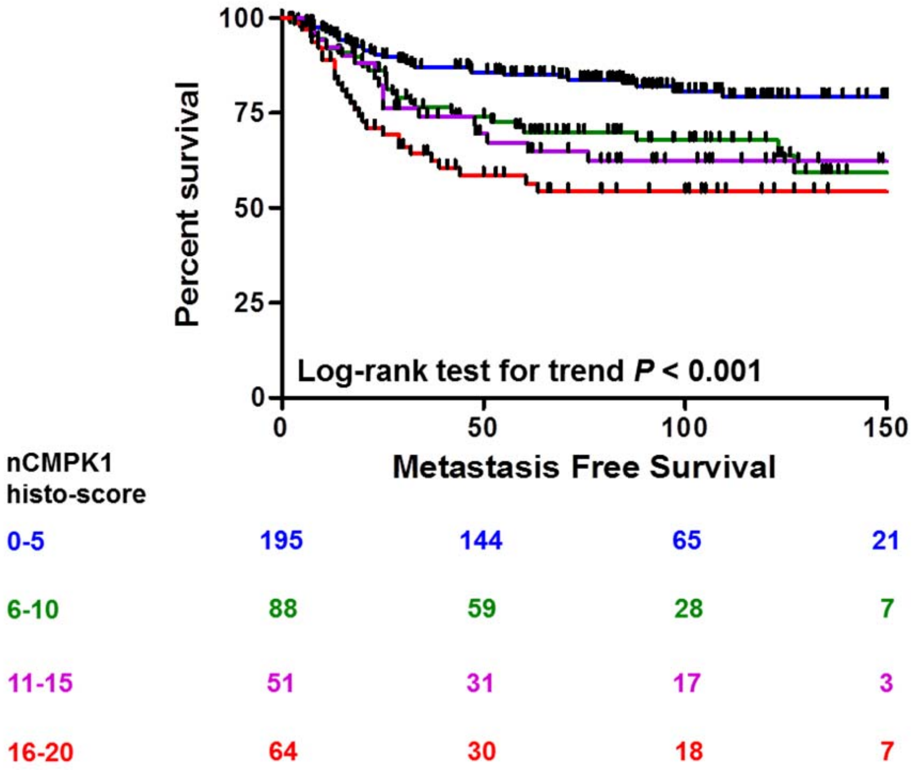

Supplement: Supplementary Information [file srep32027-s1.pdf]
